# Minimally invasive pulmonary valve replacement via left anterior mini-thoracotomy after Tetralogy of Fallot repair: a five-patient case series

**DOI:** 10.1186/s13019-026-04379-0

**Published:** 2026-06-11

**Authors:** Mohammad Abbasi Teshnizi, Behzad Alizadeh, Farhad Samadieh, Rashad Zayat, Shahram Lotfi

**Affiliations:** 1 Department of Cardiac Surgery, Imam Reza Hospital, Mashhad University of Medical Science, Mashhad, Iran; 2 Department of Cardiac Surgery, RWTH University Hospital Aachen, Pauwelsstr. 30, 52074 Aachen, Germany

**Keywords:** Pulmonary valve replacement, Tetralogy of Fallot, Minimally invasive cardiac surgery, Mini-thoracotomy, Congenital heart disease

## Abstract

**Background:**

Pulmonary regurgitation is a common long-term consequence after Tetralogy of Fallot (TOF) repair and often leads to right ventricular dilatation requiring pulmonary valve replacement (PVR). Redo sternotomy carries risks including cardiac injury and bleeding. Minimally invasive alternatives aim to reduce surgical trauma while maintaining procedural safety.

**Case presentation:**

We report five consecutive patients (aged 9–44 years) with severe pulmonary regurgitation and right ventricular dilatation who underwent minimally invasive PVR via a left anterior mini-thoracotomy (LAMT). All procedures were performed on a beating heart using femorofemoral cardiopulmonary bypass and stented bioprosthetic valves. Mean cardiopulmonary bypass time was 37.8 min. No conversions to sternotomy, major complications, or mortality occurred. Patients were extubated after a mean of 9.6 h, and mean hospital stay was 6.8 days. At two-month follow-up, all patients demonstrated excellent prosthetic valve function and significant reduction in right ventricular size.

**Conclusions:**

LAMT-PVR is a feasible and safe alternative to redo sternotomy for selected post-TOF patients. This approach provides direct access to the pulmonary artery with reduced surgical trauma and favorable early outcomes.

## Background 

Pulmonary regurgitation (PR) is a frequent late complication after Tetralogy of Fallot (TOF) repair and often results in progressive right ventricular (RV) dilatation, exercise intolerance, arrhythmias, and eventual need for pulmonary valve replacement (PVR) [1, 2]. Redo median sternotomy, the conventional approach for PVR, carries significant risks including cardiac injury, conduit disruption, bleeding, and prolonged operative time [3, 4]. Minimally invasive approaches have emerged as alternatives that may reduce surgical morbidity while providing adequate exposure for valvular intervention [5, 6].

Among these, the left anterior mini-thoracotomy (LAMT) provides a direct route to the right ventricular outflow tract (RVOT) and main pulmonary artery (MPA) [5, 6]. Early reports suggest this technique may be particularly advantageous in reoperative congenital patients, where midline adhesions pose substantial hazards [4, 5]. This case series presents our experience performing beating-heart [7] PVR using LAMT in five post-TOF patients.

## Case presentation

### Patient selection and preoperative evaluation

**Fig. 1:**
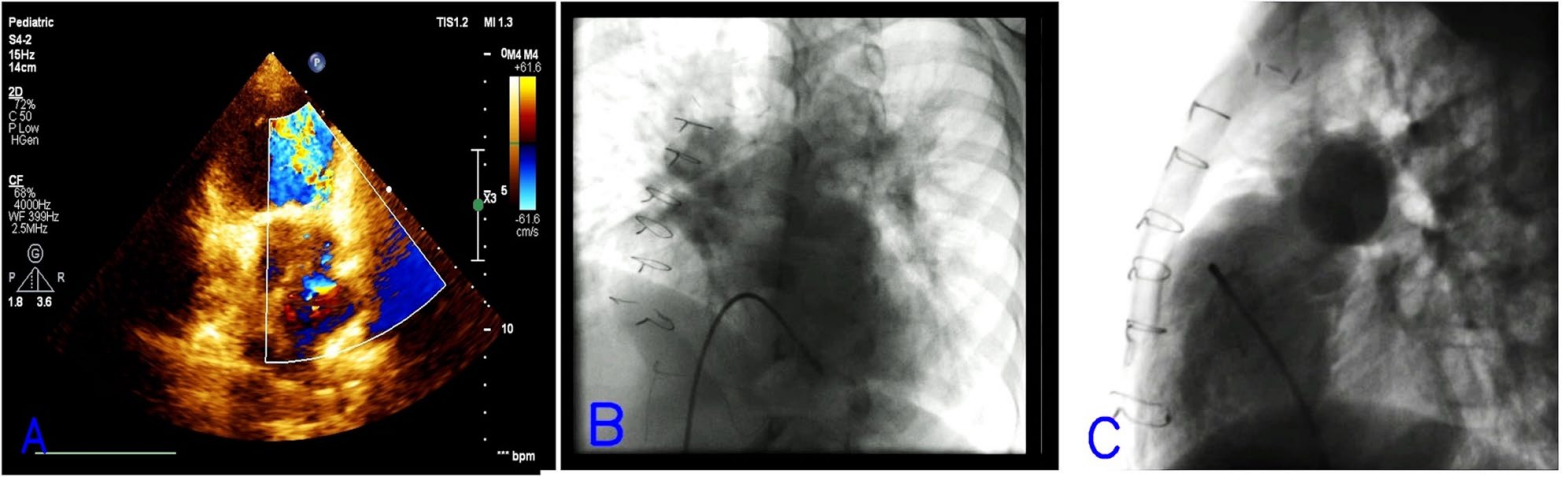
Preoperative diagnostic imaging. (**A**) Echocardiography showing a severe regurgitant jet indicating free pulmonary insufficiency. (**B, C**) Anteroposterior and lateral cardiac catheterization views demonstrating marked right atrial and ventricular dilatation

Five consecutive patients (three males, two females; mean age 28.8 years, range 9–44 years) with prior transannular-patch TOF repair underwent pulmonary valve replacement via left anterior mini-thoracotomy for severe PR and significant RV dilatation (Fig. 1; Table 1). No relevant comorbidities were present in this cohort. Inclusion criteria were repaired TOF, severe PR with RV dilatation, favorable RVOT/MPA anatomy on CT, and femoral artery diameter ≥ 5 mm on duplex ultrasound. Exclusion criteria included unfavorable retrosternal anatomy, concomitant intracardiac lesions, severe peripheral vascular disease, or the need for extensive PA reconstruction.

**Table 1:** Baseline patient characteristics and preoperative imaging findings

Case	Age (years)	Gender	Interval from initial TOF repair	Preoperative NYHA class	Pulmonary insufficiency	RV dilatation	CPB time (min)	ICU stay (days)	Hospital stay (days)
1	9	Female	11 years	II	Severe (+++)	Severe (+++)	36	2	6
2	12	Male	15 years	III	Severe (+++)	Severe (+++)	34	2	6
3	44	Male	11 years	II	Severe (+++)	Severe (+++)	40	2	7
4	41	Male	14 years	II	Severe (+++)	Severe (+++)	38	2	7
5	38	Female	13 years	III	Severe (+++)	Severe (+++)	41	2	8

TOF, tetralogy of Fallot; PR, pulmonary regurgitation; RV, right ventricle/right ventricular; RVOT, right ventricular outflow tract; MPA, main pulmonary artery; CT, computed tomography; MRI, magnetic resonance imaging; CPB, cardiopulmonary bypass; ICU, intensive care unit

Preoperative evaluation included transthoracic echocardiography to assess pulmonary valve insufficiency and RV size and function, contrast-enhanced CT to evaluate RVOT/MPA anatomy and its relationship to the left chest wall and sternum, and bilateral femoral Doppler ultrasonography to confirm suitability for peripheral cannulation. Cardiac catheterization was performed selectively to assess right-sided hemodynamics and to exclude additional abnormalities when clinically indicated.

### Surgical technique

All procedures were performed via a left anterior mini-thoracotomy through the second intercostal space on a beating heart using femorofemoral cardiopulmonary bypass and vacuum-assisted venous drainage. Patients were positioned supine with slight elevation of the left hemithorax, with the left arm tucked and the head turned slightly to the right. The relatively short cardiopulmonary bypass times reflect the limited dissection required with this minimally invasive approach.

After initiation of cardiopulmonary bypass, only a limited pericardiotomy was performed directly over the main pulmonary artery and proximal right ventricular outflow tract, thereby largely avoiding the dense retrosternal adhesions typically encountered during redo sternotomy. Exposure was optimized using vacuum-assisted drainage, gentle traction on the main pulmonary artery with a malleable retractor, and temporary reduction of ventilation or short apnea during critical steps. Dense adhesions of the anterior lung lobe were carefully lysed while protecting the phrenic nerve.

The main pulmonary artery was opened longitudinally. The aneurysmal and dyskinetic anterior RVOT tissue from the prior transannular repair was resected, and the stented bioprosthetic pulmonary valve was sewn directly onto the muscular RVOT, ensuring that no non-contractile tissue remained beneath the prosthesis. Particular attention was paid to axial alignment of the prosthetic valve with the RVOT–main pulmonary artery axis. Final positioning was confirmed visually and with transesophageal echocardiography to ensure optimal orientation and hemodynamic performance.

**Fig. 2:**
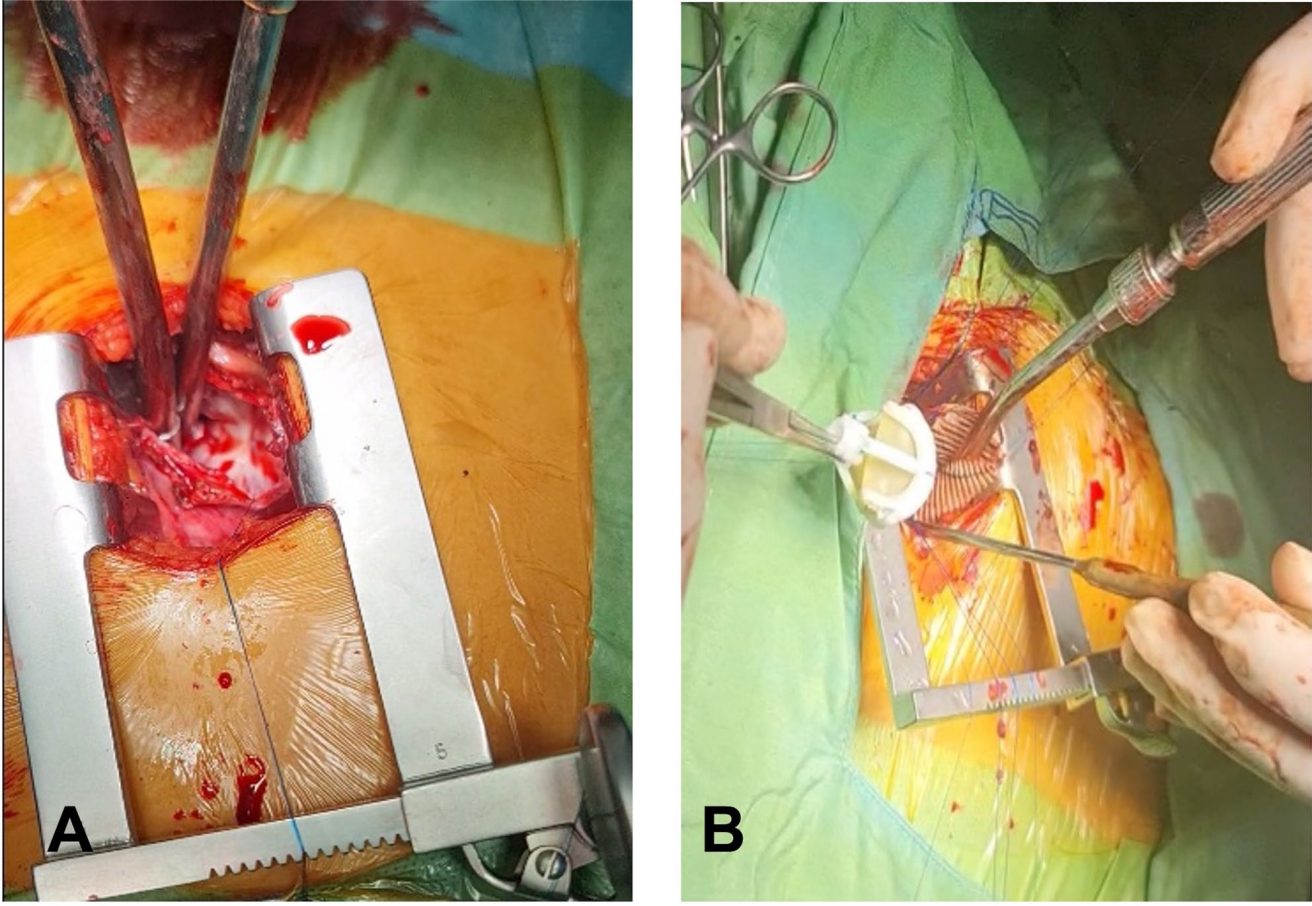
Schematic illustration of the surgical anatomy via left anterior mini-thoracotomy. Upper panel: View of the right ventricle (RV) and superior vena cava (SVC) after limited pericardiotomy. Lower panel: The aneurysmal transannular patch is resected and the pulmonary valve annulus is exposed. The bovine pericardial patch used for anterior reconstruction of the main pulmonary artery is shown (labelled “PATCH”). The prosthetic valve is seated directly onto contractile RVOT muscle

Following valve implantation, the anterior wall of the main pulmonary artery was reconstructed using a bovine pericardial patch to close the pulmonary arteriotomy (Fig. 2). No patch material was used to enlarge the RVOT or pulmonary annulus. The second intercostal space provided adequate exposure in all patients; the third intercostal space was not required in this series. Carbon dioxide insufflation was not routinely used, as satisfactory de-airing was achieved under direct vision and transesophageal echocardiographic guidance. Full readiness for immediate conversion to median sternotomy was maintained throughout the procedure, although conversion was not required in any patient. Potential challenges of this approach include limited exposure, restricted visualization, and vascular access–related complications. These risks were mitigated by meticulous preoperative imaging, optimized exposure techniques, and full readiness for immediate conversion to median sternotomy if required.

Figure 2 shows the schematic anatomy and key steps of the procedure.

### Postoperative and follow-up data

All cases were completed without conversion to sternotomy, intraoperative complications, mortality, wound infection, or reoperation for bleeding. Mean CPB time was 37.8 min. Mean time to extubation was 9.6 h, ICU stay averaged 48 h (2 days), and mean hospital stay was 6.8 days. At two-month follow-up, prosthetic valve function was normal in all patients (no paravalvular leak, mean gradient < 10 mmHg). Right ventricular size was reduced in every case (RV end-diastolic diameter ↓12–22%, RV end-diastolic area ↓15–28% by echocardiography; supplemented by MRI in three patients and CT in one). Functional status improved in all patients. Follow-up data are summarized in Table 2.

**Table 2:** Operative, postoperative, and follow-up outcomes

Case	Prosthetic valve function	Paravalvular leak	Mean gradient (mmHg)	RV size reduction	NYHA class at follow-up
1	Normal	None	8	18%	I
2	Normal	None	7	15%	I
3	Normal	None	9	22%	I
4	Normal	None	6	12%	I
5	Normal	None	8	20%	I

CPB, cardiopulmonary bypass; ICU, intensive care unit; RV, right ventricle/right ventricular; MRI, magnetic resonance imaging; CT, computed tomography

**Fig. 3:**
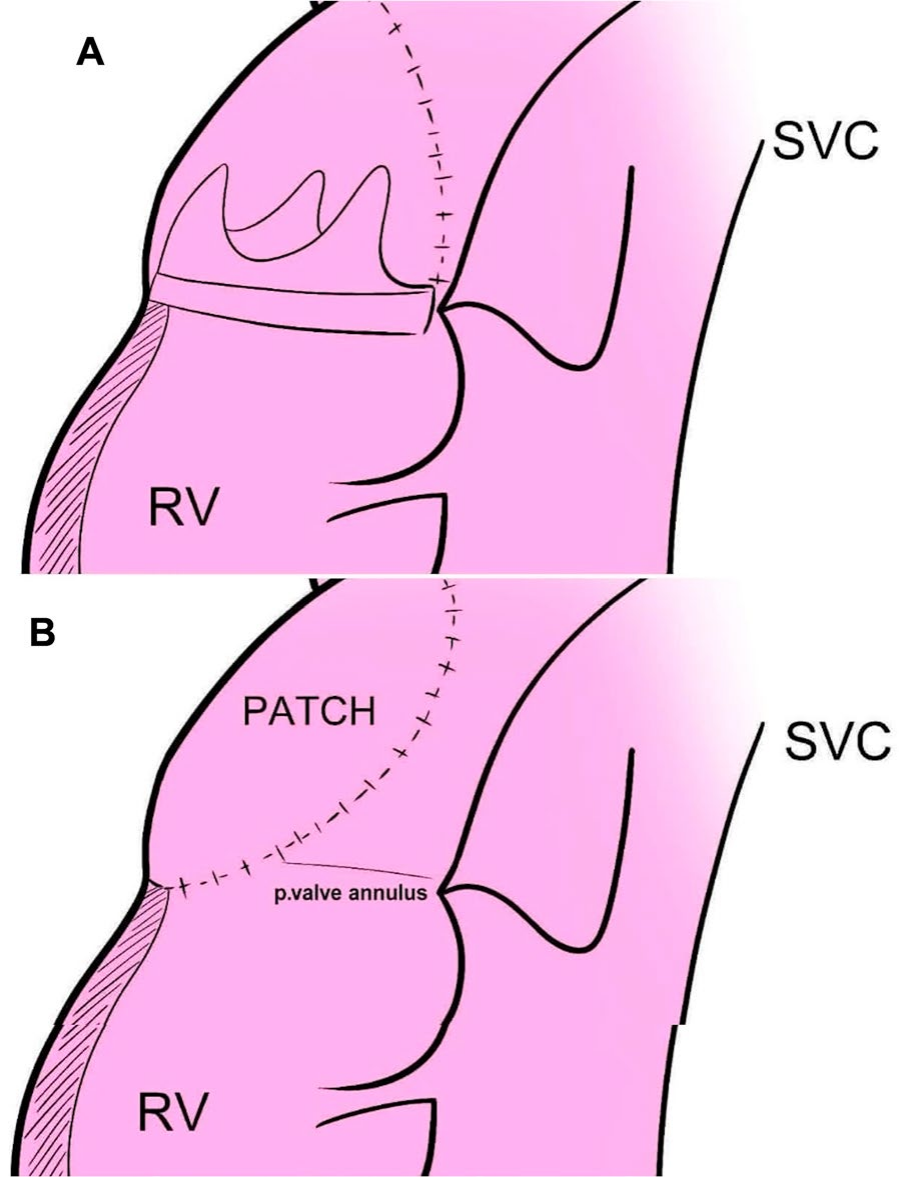
Intraoperative photographs during left anterior mini-thoracotomy pulmonary valve replacement. (**A**) Exposure of the RVOT and main pulmonary artery with femoral cannulas in place. (**B**) Resection of the aneurysmal transannular patch and preparation of the valve landing zone. (**C**) Placement of the stented bioprosthetic valve on the beating heart. (**D**) Final view after anterior main pulmonary artery reconstruction with bovine pericardial patch

Figure 3 shows the intraoperative photographs.

## Discussion

This series demonstrates the feasibility and safety of performing PVR through a left anterior mini-thoracotomy in selected patients following TOF repair [5, 6, 8]. Redo sternotomy introduces substantial risk due to adhesions between the sternum and cardiac structures, whereas LAMT provides direct, unobstructed access to the RVOT and MPA [4, 5]. Furthermore, the beating-heart technique avoids myocardial ischemia and simplifies operative management [7].

Challenges of the minimally invasive approach included restricted working space and management of adhesions from prior sternotomy. These risks were mitigated by strict patient selection based on CT anatomy, limited pericardiotomy only over the MPA/RVOT, vacuum-assisted drainage, and continuous TEE guidance. The learning curve was manageable; all procedures were performed by a surgeon with extensive experience (> 200 sternotomy PVRs).

Our findings—short CPB times, rapid postoperative recovery, and absence of major morbidity—align with previously published reports evaluating minimally invasive PVR approaches [5, 6, 8]. Reduced surgical trauma and favorable early hemodynamic outcomes underscore the potential advantages of this technique. However, limitations include the small sample size and short follow-up duration. Larger multicenter studies are necessary to validate long-term durability and refine patient selection criteria.

## Conclusions

Minimally invasive pulmonary valve replacement via left anterior mini-thoracotomy is a safe and effective alternative to redo sternotomy in appropriately selected post-TOF patients. Early outcomes demonstrate excellent feasibility, low morbidity, and promising hemodynamic results. Further studies are needed to assess long-term durability and broader applicability.

## Data Availability

Data are available from the corresponding author upon reasonable request.

## Abbreviations

CPB: Cardiopulmonary bypass
CT: Computed tomography
ICU: Intensive care unit
LAMT: Left anterior mini-thoracotomy
MPA: Main pulmonary artery
PR: Pulmonary regurgitation
PVR: Pulmonary valve replacement
RV: Right ventricle / right ventricular
RVOT: Right ventricular outflow tract
TOF: Tetralogy of Fallot
